# Clinical and laboratory features that discriminate dengue from other febrile illnesses: a diagnostic accuracy study in Rio de Janeiro, Brazil

**DOI:** 10.1186/1471-2334-13-77

**Published:** 2013-02-08

**Authors:** Regina P Daumas, Sonia RL Passos, Raquel VC Oliveira, Rita MR Nogueira, Ingebourg Georg, Keyla BF Marzochi, Patrícia Brasil

**Affiliations:** 1 Germano Sinval Faria Teaching Primary Care Center/National School of Public Health, Oswaldo Cruz Foundation – Fiocruz, Rio de Janeiro, 21041-210, Brazil; 2 Laboratory of Clinical Epidemiology/Evandro Chagas Clinical Research Institute, Oswaldo Cruz Foundation – Fiocruz, Rio de Janeiro, 21040-361, Brazil; 3 Flavivirus Laboratory, Department of Virology, Instituto Oswaldo Cruz/FIOCRUZ, 21040-190, Rio de Janeiro, Brazil; 4 Immunology Service/Evandro Chagas Clinical Research Institute, Oswaldo Cruz Foundation – Fiocruz, Rio de Janeiro, 21040-361, Brazil; 5 Laboratory of Acute Febrile Illnesses/Evandro Chagas Clinical Research Institute, Oswaldo Cruz Foundation – Fiocruz, Rio de Janeiro, Brazil

**Keywords:** Dengue/diagnosis, Signs and symptoms, Sensitivity and specificity, Fever/diagnosis

## Abstract

**Background:**

Dengue is an acute febrile illness caused by an arbovirus that is endemic in more than 100 countries. Early diagnosis and adequate management are critical to reduce mortality. This study aims to identify clinical and hematological features that could be useful to discriminate dengue from other febrile illnesses (OFI) up to the third day of disease.

**Methods:**

We conducted a sectional diagnostic study with patients aged 12 years or older who reported fever lasting up to three days, without any evident focus of infection, attending an outpatient clinic in the city of Rio de Janeiro, Brazil, between the years 2005 and 2008. Logistic regression analysis was used to identify symptoms, physical signs, and hematological features valid for dengue diagnosis. Receiver-operating characteristic (ROC) curve analyses were used to define the best cut-off and to compare the accuracy of generated models with the World Health Organization (WHO) criteria for probable dengue.

**Results:**

Based on serological tests and virus genome detection by polymerase chain reaction (PCR), 69 patients were classified as dengue and 73 as non-dengue. Among clinical features, conjunctival redness and history of rash were independent predictors of dengue infection. A model including clinical and laboratory features (conjunctival redness and leukocyte counts) achieved a sensitivity of 81% and specificity of 71% and showed greater accuracy than the WHO criteria for probable dengue.

**Conclusions:**

We constructed a predictive model for early dengue diagnosis that was moderately accurate and performed better than the current WHO criteria for suspected dengue. Validation of this model in larger samples and in other sites should be attempted before it can be applied in endemic areas.

## Background

Dengue is an acute febrile illness caused by an arbovirus (arthropod-borne virus) transmitted mainly by *Aedes aegypti* mosquitoes. Four dengue virus serotypes (DENV-1 to 4) are recognized and a primary infection by one serotype does not provide immunity against the others [1]. Nowadays, dengue is a global public health problem. It is endemic in more than 100 countries where 50 to 100 million infections are estimated to occur each year [2].

In the region of Americas, Brazil alone accounted for more than 60% of reported cases from 2000 to 2007, when epidemics occurred in different regions of the country related to the co-circulation of DENV-1, DENV-2, and DENV-3 [3]. The state of Rio de Janeiro was the site of introduction and later dissemination of these serotypes after epidemics in the years 1986, 1990, and 2002 [4]. In 2007/2008, DENV-2 was reintroduced in this state and caused an epidemic with more severe clinical presentations and more fatalities than previous ones, primarily among children and adolescents [3]. Recently, DENV-4 reemerged in the country, being identified in the state of Rio de Janeiro in 2011 [5].

Clinical presentation of dengue fever varies along a wide spectrum of signs and symptoms. Typically, it presents as a self-limiting disease characterized by fever associated with symptoms such as headache, nausea, vomiting, arthralgia, myalgia and/or rash. However, during the course of the disease, some patients develop severe manifestations related to increased vascular permeability and plasma leakage that can lead to death. Signs of spontaneous bleeding may be present and are more frequent in severe forms [6]. There is no specific therapy, but timely initiated supportive treatment can reduce the lethality of severe cases to less than 1% [7].

Early identification of dengue infection could help clinicians to institute adequate case management and to identify patients who should be closely monitored for signs of plasma leakage. This information might promote early supportive therapies [8], prevent the use of potentially harmful drugs [9] and encourage assessment of prognosis and the use of treatment guidelines [10].

Despite the recent development of rapid laboratory tests [11-13], their availability is still limited and most diagnoses in endemic areas are based on clinical and epidemiological criteria. Until 2006, most studies on clinical features to discriminate dengue from other febrile illnesses have evaluated hospitalized patients in Southeast Asia [14]. Despite some evidence that clinical signs and symptoms vary during the course of the disease, few of those studies reported disease duration at the time of clinical evaluation. Therefore, comparison of results and identification of useful features for early diagnosis was difficult [14].

In recent years, the focus on early clinical predictors [15,16] and the use of regression methods to evaluate multiple predictors has increased [15-17]. Some studies using these methods were also done in the Americas [18-21]. In Brazil, although some studies have identified signs and symptoms associated with confirmed dengue cases [22,23], the evaluation of multiple clinical criteria with the aim of constructing a diagnostic algorithm has not yet been attempted.

This study aims to: 1) identify clinical and laboratory features useful for discriminating dengue from other febrile illnesses (OFI) in the first three days of disease; and 2) evaluate the accuracy of combinations of these features.

## Methods

### Settings and participants

A sectional diagnostic study was conducted with data that were systematically collected at the outpatient clinic for Acute Febrile Illnesses (AFI) of a public research institute from January 2005 to July 2008. Data collected once a week by the same staff in the Emergency Department (ED) of a public general hospital from November 2007 to January 2008 were also included. Patients older than 12 years of age who presented with a history of fever lasting up to three days without an evident or suspected focus on clinical examination (such as tonsillitis or pyelonephritis) were eligible to be included in the study. Those with severely compromised health status (i.e. with altered consciousness, signs of shock or severely dehydrated) in need of emergency care were excluded. Enrollment was sequential at the outpatient clinic, four afternoons a week. At the emergency department, once a week the research team evaluated the first eight eligible patients who attended the ED. Both health units are located in the city of Rio de Janeiro and the research institute does not provide pediatric care. Although dengue fever is considered endemic in Rio de Janeiro, from November 2007 to April 2008 a DEN-2 epidemic was also detected in the city.

### Collection of patient data

Attending staff included two infectious disease specialists and two supervised medical students. All were trained to perform and record standardized clinical evaluations and fill a form containing 28 symptoms and 25 signs potentially useful in the differential diagnosis of febrile illnesses, according to literature review and specialists consensus. Interobserver agreement on signs and symptoms was evaluated in a subsample of 140 patients and was almost perfect (kappa > 0.80) for most collected clinical data [24]. The questionnaire is available online for consultation and details on its development have been described elsewhere [24].

On initial presentation, patients were evaluated through comprehensive clinical history taking and physical examination. Clinical data were recorded on the standardized form and blood samples were collected for a complete blood count and dengue diagnostic tests. A second sample was requested for all patients 7–10 days after the initial visit for paired serology. Additional laboratory tests were done according to individual clinical judgment.

The study was approved by the IPEC-FIOCRUZ Ethics Committee and all the patients and/or their guardians agreed to participate by providing a written informed consent.

### Variable definitions

Clinical variables investigated were standardized according to clear definitions described in the research protocol and included the following: hepatomegaly – liver size of 12 cm or more at percussion; lymph node enlargement – presence of one or more lymph nodes measuring 1 cm or more at palpation; pallor – any degree of skin or mucosa paleness; dehydration – dry mucosa at inspection; and splenomegaly – a palpable spleen at inspiration with the patient lying on his right side. Taste disorder was considered present when patients answered affirmatively to the question “Does food or water taste differently?”

### Laboratory methods

Acute and convalescent serum samples were tested for anti-dengue immunoglobulin M (IgM). Blood samples collected up to the fifth febrile day were also screened for viral RNA using reverse-transcriptase polymerase chain reaction (RT-PCR) and/or tested for NS1 antigen. Light microscopy of thick stained blood smears for intracellular malarial parasites was performed in acute blood samples from patients who had been in malaria endemic regions.

Tests for IgM detection were done using an IgM antibody-capture enzyme-linked immunosorbent assay (MAC-ELISA) (PanBio, Brisbane, Australia) according to the manufacturer’s instructions. Dengue NS1 antigen was detected with the Platelia™ Dengue NS1Ag-ELISA (Bio-Rad Laboratories Marnes-la-Coquette, France). Both tests were performed at the Laboratory of Immunodiagnostics at IPEC-FIOCRUZ. RNA detection was performed in the Laboratory of Flavivirus, Oswaldo Cruz Institute (FIOCRUZ) using RT-PCR according to Lanciotti *et al*. [25].

### Case definitions

Dengue diagnosis was based on the combination of different tests, considering their accuracy in different disease phases [1,26-28]. Stating as day 0 the day of fever onset, we classified patients as:

Dengue (D): A patient who tests positive for either IgM antibodies, viral RNA (RT-PCR) or NS1 antigen;

Non-Dengue (ND): A patient who tests negative for anti-dengue IgM antibodies in a sample collected between day 7 and day 21 OR a patient with a unique sample collected between day 0 and day 2 that is negative for both NS1 antigen and anti-dengue IgM;

Indeterminate: A patient who is negative for IgM antibodies and NS1 antigen in an acute sample collected after day 2 for whom a convalescent sample is not available.

### Statistical analysis

We carried out an exploratory analysis of clinical and laboratory features comparing D and ND groups. We used Pearson Chi-square or Fisher’s exact test for categorical and Mann-Whitney test for continuous variables, considering a level of significance of 0.05. Variables that were significant in the exploratory analysis were selected for inclusion in a multiple logistic regression model. The two estimated models included: a) clinical data only, and b) clinical and laboratory data.

Logistic regression parameters were estimated with the Firth procedure that uses the penalized likelihood function [29]. This method enables estimation of parameters in small samples with strongly predictive covariates, where the phenomenon of separation makes at least one parameter estimate diverge to ± infinity. A manual stepwise backward selection approach was used, considering as the criterion for maintaining variables in the model a p-value of less than 0.05 on the penalized log-likelihood ratio test.

For the regression model including laboratory data, a receiver-operating characteristic (ROC) curve was plotted. The best cut-off value for probabilities was determined by the Youden index that maximizes the sum of sensitivity and specificity. Subsequently, these values were substituted in the model’s equation and the combinations of variable values that classified a patient as a dengue case were determined.

We also evaluated the predictive accuracy of the WHO criteria for probable dengue, which states that at least two of the following manifestations must be present in an acute febrile patient: headache, retro-orbital pain, myalgia, arthralgia, exanthema, leukopenia, and hemorrhagic manifestations [30]. For each patient, we computed the number of these manifestations (from zero to seven) that were present. We defined leukopenia as a leukocyte count lower than 4,000/mm^3^.

With the aim of ascertaining the best predictive model, ROC curve analysis was also done for WHO criteria and the difference between the areas under the curves (AUC) of the curves’ pair (estimated model x WHO criteria) was tested by Delong’s method [31]. Confidence intervals (CI) for the AUC, sensitivities and specificities were estimated using pROC package functions which compute the 95% CI with 2000 stratified bootstrap replicates (with replacement) [32]. Data collected in forms were entered using Epidata 3.1 software [33]. Exploratory analyses were done with Statistical Package for Social Sciences - SPSS v. 17.0 (SPSS Inc., Chicago, Illinois). Logistic regression with Firth correction was implemented with *logistf* package [34] and ROC curve analyses with *pROC* package [32] from the R 2.11.1 software [35].

## Results

From a total of 182 evaluated patients, 40 (22.0%) were excluded from analyses as their diagnoses were indeterminate. From the remaining 142 patients, 69 were classified as dengue (D) and 73 as non-dengue (ND). Median age and distribution by health units were not different between D and ND patients. Male sex was predominant (65.2%) among dengue cases, but was not among non-dengue patients (47.9%). The majority of dengue cases (71%) were enrolled in the epidemic period (Table 1). DENV-3 was the unique identified serotype in the first years of the study, whereas DENV-2 was firstly isolated in January 2008 and became the predominant serotype since then (data not shown).

**Table 1:** **Characteristics of the sample according to diagnosis of dengue or non**-**dengue**

	**Dengue**		**Non**-**dengue**		**p value**^**a**^
	(**N** = **69**)		(**N** = **73**)		
Sex, n (%)					
Male	45	(65.2)	35	(47.9)	0.028
Female	24	(34.8)	38	(52.1)	
Age in years, median (IR)	31.0	(23–41)	33.2	(24–39)	NS
Period, n (%)					
Epidemic	49	(71.0)	37	(50.7)	0.010
Endemic	20	(29.0)	36	(49.3)	
Health unit, n (%)					
IPEC	62	(89.9)	62	(84.9)	NS
HMLJ	7	(10.1)	11	(15.1)	

NOTE. – ^a^ Chi-square test for categorical variables and Mann-Whitney test for continuous variables; IR = Interquartile range.

The most frequent clinical features in dengue patients were exhaustion (98.6%), myalgia (92.8%), and headache (87.0%). However, these features were also very prevalent among ND patients and were useless for discriminating between D and ND patients. From clinical history, only taste disorder (OR: 2.15; IC95%:1.06–4.36) and the report of rash (OR: 3.33; IC95%: 1.56–7.07) were associated with dengue diagnosis. Bitter taste and diminished taste were the most frequently reported taste disorders. On physical examination, rash, lymph node enlargement, and conjunctival redness were significantly more frequent in D patients than in ND patients (Table 2). Considering laboratory results, platelet and leukocyte counts were significantly smaller in the D than in the ND group (Table 3).

**Table 2:** Clinical features associated to dengue diagnosis in patients evaluated up to 3 days after fever onset

**Clinical data**	**Dengue**		**Non-dengue**		**OR(95%CI)**
	**N**	**%**	**N**	**%**	
History:					
Exhaustion	68	98.6	69	95.8	
Myalgia	64	92.8	67	93.1	
Headache	60	87.0	64	87.7	
Lumbar pain	59	85.5	59	81.9	
Anorexia	55	79.7	55	75.3	
Retro-orbital pain	49	71.0	44	60.3	
Taste disorder	47	70.1	36	52.2	2.15 (1.06–4.36)
Nausea	45	65.2	40	54.8	
Arthralgia	44	63.8	43	58.9	
Photophobia	38	55.9	35	47.9	
Chills	33	47.8	42	57.5	
Dizziness	33	47.8	34	46.6	
History of rash	30	44.1	14	19.2	3.33 (1.56–7.07)
Cough	25	36.2	26	35.6	
Diarrhea	25	36.2	18	24.7	
Vomiting	22	31.9	17	23.6	
Itching	21	31.3	13	18.6	
Abdominal pain	21	30.4	31	42.5	
Coryza	20	29.0	21	28.8	
Sore throat	16	23.2	27	37.0	
Nasal congestion	14	20.9	17	24.6	
History of bleeding	12	17.4	6	8.2	
Earache	11	16.2	5	6.9	
Hoarseness	11	16.2	9	12.5	
Dyspnea	10	14.5	7	9.7	
Physical exam:					
Rash	48	71.6	31	45.6	3.02 (1.48–6.16)
Conjunctival redness	38	55.9	20	29.0	3.10 (1.53–6.29)
Lymph node enlargement	33	50.8	14	20.6	3.98 (1.86–8.53)
Dehydration	24	35.3	16	23.2	
Pharyngeal erythema	15	22.4	21	31.3	
Pallor	11	16.4	15	21.7	
Petechiae	9	13.4	4	5.9	
Hepatomegaly	7	10.4	5	7.4	
Splenomegaly	5	7.5	5	7.2	

Crude odds ratios (OR) and respective 95% confidence intervals (CI) are shown for those with statistical significance.

**Table 3:** **Clinical signs and hematologic parameters according to definite diagnosis** (**dengue or non**-**dengue**)

	**Median (IR)**		**p value**^**a**^
	**Dengue**	**Non**-**dengue**	
Axillary temperature (°C)	37.1	37	NS
	(36.5–38.2)	(36.5– 37.8)	
Heart rate	84	80	NS
	(78.0–90.0)	(72.0–91.0)	
Respiratory rate	19	19	NS
	(16.0–20.0)	(16.0–20.0)	
MAP sitting (mmHg)	92.5	88.3	NS
	(83.3–100.0)	(77.5–96.7)	
MAP recumbent (mmHg)	93.3	93.3	NS
	(83.3–102.5)	(83.3–97.9)	
Hematocrit (%)	42	42.3	NS
	(39.3–45.0)	(40.0–45.1)	
Platelets (1000/mm^3^)	175	209	0.002
	(121.5–218.0)	(171.0–237.5)	
Hemoglobin (g/dl)	14.4	14.2	NS
	(13.2–15.3)	(13.6–15.3)	
Leukocytes (/mm^3^)	3600	6205	< 0.001
	(2745–5015)	(4248–8718)	

NOTE. – IR = Interquartile range. ^**a**^ Non-parametric Mann-Whitney test. MAP = Mean arterial pressure.

The variables that were significantly associated with dengue diagnosis in the univariate analysis were selected for inclusion in multiple regression models. These models were based on data from 97 patients (52 D and 45 ND) as the others had incomplete data for one or more of the selected covariates. The results of multiple regression analyses are presented in Table 4. In the model including clinical data only (Model 1), conjunctival redness and history of rash were the variables independently associated with the diagnosis. At the best cut-off value, it showed an 84.6% sensitivity and 66.7% specificity. According to the model equation, this accuracy is achieved when we classify patients with conjunctival redness or exanthema as D and those with neither of these features as ND.

**Table 4:** **Adjusted odds ratios and accuracy parameters for dengue diagnosis of multiple logistic regression models according to the range of included data** (**clinical only or clinical and laboratory**)

	**Adjusted diagnostic odds ratio**		**Sensitivity**		**Specificity**	
	**OR**	**95%CI**	**%**	**95%CI**	**%**	**95%CI**
Model 1 - Clinical data only						
History of Rash	3.84	1.54–10.24	84.6	46.8–93.0	66.7	38.6–78.8
Conjunctival redness	4.05	1.69–10.31				
Model 2 - Clinical and laboratory data						
Conjunctival redness	4.1	1.61–11.22	80.8	59.6–94.6	71.1	53.3–84.4
Leukocytes (ln)^a^	0.13	0.04–0.34				

NOTE. – OR: odds ratio; 95% CI: 95% confidence interval. ^a^ Effect for each unit of natural logarithm of leukocyte count.

**Figure 1:**
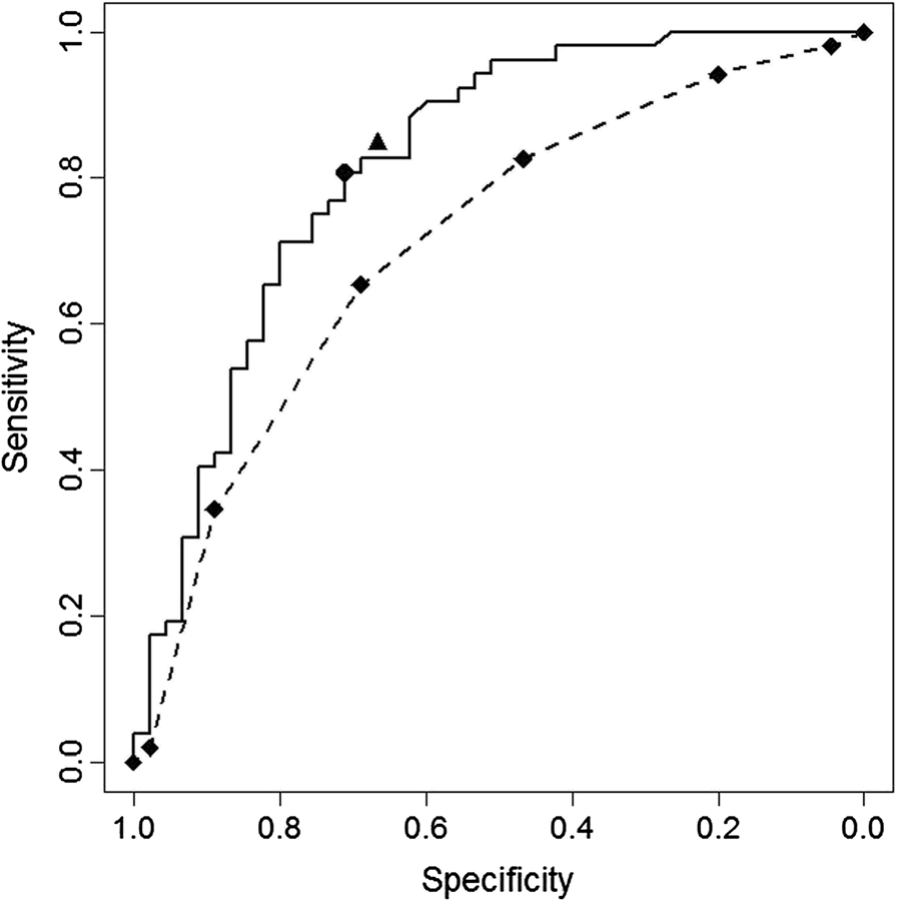
**Receiver**-**operating characteristic** (**ROC**) **curves for the logistic model with clinical and laboratory data** (**Logistic**) **and for the WHO criteria for probable dengue** (**WHO).** Sensitivity and specificity at the best cut-off points for the models with clinical and laboratory data (●) and clinical data only (▲). ROC Curves (Area; 95% CI)_____ Logistic (0.82; 0.73–0.90); _ _ _ _ WHO (0.71; 0.61–0.81).

In the logistic regression with clinical and laboratory data, only conjunctival redness and leukocyte count showed to be independently associated with the diagnosis and were selected to remain in the final model (Model 2). This model showed an AUC ROC of 0.82 with 80.8% sensitivity and 71.1% specificity at the best cut-off value. Adjusted odds ratios for each model variable are shown on Table 4. WHO criterion for probable dengue—the presence of two out of seven features—showed 98.0% sensitivity but only 4.4% specificity. The ROC curve for the WHO criteria showed an AUC of 0.71, which was significantly smaller than that of Model 2 (p = 0.04) (Figure 1).

Replacing the best cut-off value in the Model 2 equation, we found two combinations of variable values that would indicate a dengue diagnosis in this setting: 1) conjunctival redness in a patient with a leukocyte count below 7,500/mm^3^; or 2) leukocyte counts below 3,760/mm^3^ independently of other features. 

## Discussion

Dengue is one of the most underreported tropical diseases [1]. On the other hand, during epidemics, overreporting can occur at some health sites. The lack of laboratory resources and the nonspecific clinical presentation of non-severe cases greatly contribute to this situation [36]. Diagnostic algorithms based on clinical data may enhance disease diagnosis and surveillance in endemic areas.

In this study, we identified some clinical and hematological features associated to confirmed dengue cases. We also constructed simple predictive models for dengue diagnosis based on clinical and hematological data for patients presenting early in the course of disease. These models showed moderate accuracy [37], and performed better than the diagnostic features proposed by WHO for probable dengue [30] in the study population.

Among clinical features, rash, taste disorder, conjunctival redness, and lymph node enlargement were all associated with dengue diagnosis. Rash is among the classic signs of dengue fever and its description includes different cutaneous manifestations, such as a diffuse erythema coincident with fever or a macular exanthema that is more frequent after the third day of disease [1,6]. Rash has also been reported to be more common in primary than in secondary infections [38], an issue that we could not explore in our study.

The extraordinarily high prevalence of rash detected on the physical exam of dengue patients (72%) in our study can be related to major efforts to ascertain any kind of cutaneous manifestations in a research setting. These may have included a mild flushing that would otherwise be unnoticed. It should be noted that a history of rash was more accurate for dengue diagnosis than its detection on physical exam. The same finding was also reported by Chadwick *et al*. [17] who found that history of rash was the only independent clinical predictor that remained in a regression model which included clinical and laboratory data. In this context, the possibility that self-examination could be more accurate than medical examination at ascertaining some mild cutaneous manifestation should be considered.

Comparing our results with other reports, we found that taste alteration, conjunctival redness, and lymphadenopathy, although described as common manifestations of dengue fever [1], are not consistently investigated as predictive signs and symptoms in studies on dengue diagnosis [14]. Nevertheless, taste alteration was reported as a dengue predictor by the few researchers who investigated this symptom [15,39], and conjunctival redness was reported by Low *et al*. [15] as one of the most accurate signs (p < 0.0005; OR = 4.49) for dengue diagnosis among adult outpatients with a fever lasting less than 72 hours. The frequency of lymph node enlargement in dengue patients has varied from 3% [14] to about 20% [17,23] in studies that reported this sign. In these studies, it was not a useful sign to discriminate D from ND patients, despite being more frequent among the first ones in all of them.

The importance of ocular findings warrants better investigation as recent studies have described a variety of ocular manifestations in dengue [40-42], suggesting that eye involvement may be more common than usually appreciated. Gregory et al. (2010) also highlighted the importance of ocular manifestations in dengue as they found retro-orbital pain as an important clinical feature to discriminate dengue from OFI in all age groups [20].

Among hematological data, leukocyte count was the most discriminant feature. Although common in other viral illness, leukopenia has been consistently reported as an independent predictor of dengue diagnosis among febrile patients [14,16,19], particularly in adults [19,20,43-46], and seems to occur earlier than thrombocytopenia [43,44]. Indeed, it was the most important isolated predictor in our study.

Other authors have already proposed models and scales to predict dengue infection in febrile adults living in the Americas. Comparison between predictive rules and WHO criteria through ROC curve analysis was also done by some of them. In Colombia, Díaz et al. (2006) developed a scale which performed better as an early predictor in adults than the one produced with WHO criteria (AUC ROC of 81.0% versus 70.0%). It was composed by the presence of rash, positive tourniquet test, absence of nasal discharge, arthralgias, absence of diarrhea (1 point for each finding), leukocyte count <4,000/mm^3^ (3 points) and platelet count <180.000/mm^3^ (2 points) [19]. Similar accuracy was obtained in Puerto Rico with a predictive model for adults with suspected dengue (AUC ROC: 79.7%). Retro-orbital pain, rash, absence of sore throat, and leukopenia were the independent predictors in this age group [20].

As strengths of this study, we may cite the collection of clinical data by a trained team, before the performance of laboratory tests, and the use of a comprehensive standardized protocol for history taking and examination. The recording of clinical features prior to any test result minimized the risk of observation bias, and the inclusion of clinical features other than the usual dengue signs and symptoms allowed us to identify some potentially useful new predictors.

This study also has some limitations. The first one is related to the small sample size used in multiple regression analysis. This may be attributed in part to the characteristics of our clinic, which attends mainly referred patients, to a long period without epidemics when we had slow subject enrollment and to our option to analyze only data from patients in the first three days of disease. The small sample size precluded any subgroup analyses.

We also had considerable losses because of indeterminate diagnosis (40/182), as only 66 out of 182 (36.3%) eligible patients collected two blood samples. Although the use of NS1 test helped to diagnose dengue in patients with samples collected from day 0 to day 2, those with only one negative sample collected between day 3 and day 7 were classified as indeterminate, as a negative result in both IgM and NS1 could be a false-negative by this time. As a consequence, we had relatively few confirmed ND patients. This kind of problem, however, affects most studies with outpatients, in such a way that losses greater than 30% for indeterminate diagnoses are common in this setting [20,47].

We cannot exclude the possibility of some misclassification by our reference standard in dengue diagnosis. Although sensitivities above 90% for NS1 on days 0 to 2 [26,48] and sensitivities above 93% for IgM in samples collected after the seventh day of disease have been reported in patients with a primary infection [1,28,49], both tests have lower sensitivities in secondary infections [28,50]. As dengue is endemic in Rio de Janeiro, it is possible that some dengue patients have been classified as non-dengue. The consequence of this eventual misclassification would be a decrease in the measured odds ratios with underestimation of the accuracy of analyzed predictors.

The interpretation and generalization of the results of studies such as this one must consider the fact that diagnostic accuracy of clinical manifestations also depends on their frequency in the non-dengue group. This means that it varies according to the incidence of other febrile illnesses (OFI) in the same period and place. For instance, low platelet counts may be useless to discriminate dengue from OFI in a place where malaria is endemic, as low platelet counts are also frequent in the later [17]. In our study, laboratory tests for OFI were required as indicated by clinical suspicion and we are unable to describe the prevalence of other diagnoses, except for malaria, which was rare (2%) in the study population.

Although our model outperformed the diagnostic accuracy of the WHO criteria, its predictive value is still poor, with some 20% false negative among dengue patients and 30% false positive among those with OFI. The low accuracy of WHO case definition, mainly because of its low specificity has been described by Martinez et al. (2005), who also explored the accuracy of different number of WHO criteria [51]. The difficulty to identify early clinical predictors of dengue infection in adults has been described by Ramos et al. (2009) in a large study in Puerto Rico. Although they identified eye pain, diarrhea and absence of upper respiratory symptoms as independently associated to confirmed dengue cases, they also highlighted the low predictive value of these features, alone or in combination, for early infection in adults [18]. In spite of this relatively low accuracy, the use of management protocols based on clinical diagnostic scales proved useful to reduce hospitalizations due to dengue in Colombia [52].

Nonetheless, the results of this study are potentially helpful for surveillance in adults, as they suggest that the proposed criteria, derived from simple clinical and hematological data, can be more accurate than the criteria for reporting suspected dengue cases currently in use. The use of this alternative algorithm could enhance the ascertainment of dengue cases by clinical and epidemiological criteria, and enable a more accurate estimation of the disease burden. Meanwhile, it should be stressed that in endemic areas, while early accurate laboratory tests are not widely available, dengue fever should be considered in every patient presenting with an acute undifferentiated febrile illness. Monitoring all these patients for the development of signs of severity, however, may impose a great burden on the health services. Once validated, algorithms that enable early identification of dengue cases could influence clinical outcomes as they would allow closely monitoring of selected patients. This procedure may warrant timely identification of alarm signs and the adoption of simple and widely available therapeutic support measures that are effective in preventing fatalities [7].

## Conclusions

We constructed predictive models for early dengue diagnosis that were moderately accurate and performed better than the diagnostic features proposed by WHO for dengue surveillance.

Considering only clinical features, the presence of conjunctival redness or a history of skin rash, in contrast with the absence of these features, showed 84.6% sensitivity and 66.7% specificity for dengue diagnosis. Conjunctival redness in a patient with a leukocyte count below 7,500/mm^3^ or leukocyte counts below 3,760/mm^3^ independently of other features showed an 80.8% sensitivity and 71.1% specificity.

These results can be useful for the development of predictive clinical tools as they may call attention to signs that are frequently ignored on dengue research. Validation of the proposed predictive model in larger datasets is advisable before using it for clinical or surveillance purposes. Studies with large sample sizes are also needed to identify and confirm predictors of dengue infection in different epidemiological contexts.

## Abbreviations

OFI: Other febrile illnesses; ROC: Receiver-operating characteristic; PCR: Polymerase chain reaction; DEN-V: Dengue virus; OFI: Other febrile illnesses; AFI: Acute febrile illnesses; WHO: World Health Organization; IgM: Immunoglobulin M; RNA: Ribonucleic acid; RT-PCR: Reverse-transcriptase polymerase chain reaction; MAC-ELISA: IgM antibody-capture enzyme-linked immunosorbent assay; D: Dengue case; ND: Non-dengue case; ED: Emergency department; AUC: Area under the curve.

## Competing interests

The authors declare that they have no competing interests.

## Authors’ contributions

RPD participated in the design of the study, performed the statistical analysis and drafted the manuscript. RVCO participated in statistical analysis. RMRN carried out molecular biology assays. IG carried out the immunoassays. SRLP, PB and KBFM conceived the study, participated in its design and coordination and helped to draft the manuscript. All authors revised the manuscript, contributed to its content, read and approved its final version.

## Pre-publication history

The pre-publication history for this paper can be accessed here:

http://www.biomedcentral.com/1471-2334/13/77/prepub
